# Mechanical Properties of Carbon Fiber and Polyimide Fiber Hybrid Reinforced Polyimide Resin Matrix Composites at Room and High Temperatures

**DOI:** 10.3390/polym18111322

**Published:** 2026-05-27

**Authors:** Ningqi Lu, Hongkun Gao, Yizhuo Gu, Hongtong Dou, Yibin Li

**Affiliations:** 1 Tianmushan Laboratory, Hangzhou 310023, China; luningqi2023@163.com (N.L.); bht0087@tmslab.cn (H.D.); 2 School of Materials Science and Engineering, Beihang University, Beijing 100191, China; hkgao@buaa.edu.cn

**Keywords:** PI fiber, carbon fiber, hybrid composite, high-temperature resistance

## Abstract

High-strength, high-modulus polyimide (PI) fibers are a type of high-performance organic fiber known for their exceptional high-temperature resistance. When blended with carbon fibers to prepare hybrid composite materials, they have the potential to strike a balance between rigidity and toughness, thereby offering a composite structure with high modulus, strength and high toughness. In this study, a series of hybrid fiber-reinforced composites were fabricated using high-strength, high-modulus PI fibers together with carbon fibers as reinforcements and a PI resin matrix. The effects of the hybrid ratio on the tensile, compressive and flexural properties, as well as the failure modes, were systematically investigated. Experimental results showed that, compared to pure PI fiber composites, the hybrid fiber composites exhibited significant improvements in the compressive and flexural properties, in accordance with the hybrid law. Specifically, the hybrid composites demonstrated a negative hybrid effect in terms of tensile properties, whereas they exhibited a positive hybrid effect in terms of compressive and flexural properties. In high-temperature flexural tests, the addition of carbon fibers significantly enhanced the retention of the properties at 300 °C and 370 °C; for instance, the incorporation of carbon fibers at a volume fraction of 24% enhanced the flexural strength retention rate of the composite laminate at 300 °C from 37% to 66%, and remarkably increased the modulus retention rate from 50% to 94%, showing great advantages of the hybrid composite in a load-bearing structure at elevated temperatures.

## 1. Introduction

Hybrid fiber composites are fabricated by combining two or more types of fibers, enabling the disadvantages of one fiber to be mitigated by the advantages of another, while simultaneously preserving the inherent benefits of each constituent [1,2,3]. Such hybridization contributes to a more comprehensive balance of material properties. Furthermore, the incorporation of multiple fiber types markedly enhances the design versatility of the composite. Systematic variations in fiber selection, relative proportions, and mixing architectures can generate composites with differentiated performance characteristics, thereby enabling the determination of an optimized configuration aligned with specific functional and structural requirements [4,5].

Carbon fiber composites exhibit outstanding properties, such as low density, high specific strength, and high specific modulus. Their applications in the aerospace industry have become highly advanced, with extensive utilization in structural components, including wings, empennages, hatches, and fuselage sections [6,7]. However, the inherently high strength and stiffness of carbon fibers are accompanied by intrinsic drawbacks, most notably their pronounced brittleness. These deficiencies significantly constrain impact resistance and damage the tolerance of carbon fiber composites [8]. Accordingly, hybridization with fibers of higher toughness and ductility represents a critical strategy to overcome these limitations, enabling the development of composites with improved structural resilience and broader applicability in demanding aerospace environments [9,10].

High-toughness fibers are predominantly organic, such as aramid fibers and ultra-high molecular weight polyethylene (UHMWPE) fibers, representing the most widely utilized examples of hybrid composite [11,12]. Wang et al. systematically investigated hybrid laminates reinforced with T300 carbon fiber plain-weave fabrics and aramid fiber plain-weave fabrics [13]. Their findings revealed that the tensile modulus was primarily governed by the fiber mixing ratio, while the tensile strength was strongly dependent on the stacking sequence. In particular, the laminates with higher content of carbon fiber in the central layers exhibited superior tensile strength. Flexural performance, on the other hand, was dictated largely by the fiber type in the outermost layers, with carbon fiber/aramid fiber alternately layered laminates exhibiting the poorest bending performance. Expanding on this, Chen et al. explored the thermo–mechanical behavior of carbon fiber/aramid fiber plain-weave hybrid laminates across a temperature range [14]. Their results demonstrated that a configuration with aramid fibers as the surface layers and carbon fibers as the core significantly enhanced the overall performance. The incorporation of carbon fibers improved strength and stiffness, whereas the addition of aramid fibers contributed to enhanced toughness and alleviated brittle fracture modes. Nevertheless, the tensile and flexural properties of these hybrids exhibited strong temperature dependence, with a marked decline observed as the temperature increased from 30 °C to 40 °C. Lu et al. prepared interlayer-hybrid composites consisting of UHMWPE fibers and carbon fibers, and reported that flexural strength, compressive strength, and interlaminar shear strength increased monotonically with increasing carbon fiber content [15]. Overall, these investigations provide compelling evidence that hybridization with high-toughness organic fibers can effectively mitigate the intrinsic brittleness of carbon fiber composites.

High-strength and high-modulus polyimide (PI) fiber represents a new generation of high-performance organic fibers. Beyond inherent strength and toughness of conventional organic fibers, it offers superior thermal resistance, exceptional ultraviolet (UV) radiation resistance, and outstanding thermal stability [16,17,18]. Yin et al. subjected PI fibers to stretching at various draw ratios in an oven maintained at 450 °C, resulting in a pronounced elevation of the glass transition temperature [19]. Furthermore, the temperature corresponding to 5% thermal decomposition increased by 45 °C relative to the unstretched fibers, reaching as high as 541 °C. Omid et al. prepared PI aerogel fiber with an initial decomposition temperature as high as 586 °C [20]. Jin Min et al. prepared continuous PI fiber-reinforced ceramic matrix composites, which maintained structural integrity for a long time even in flames at 1000 °C [21]. Due to its high toughness, the composite can also be mixed with carbon fiber to enhance the toughness of the composite. For example, He et al. investigated the mechanical behavior of carbon fiber/polyimide fiber-reinforced epoxy composites with varying stacking sequences and mixing ratios [22]. Their results indicated that both the tensile performance and compressive performance generally increased linearly with the mixing ratio, while the addition of PI fibers also improved the tensile fracture elongation of the composite. With respect to flexural performance, placing the carbon fibers on the compression side markedly enhanced the flexural strength, while positioning the carbon fibers on both the tensile and compression sides contributed to increased flexural modulus. Notably, a critical mixing ratio was observed for the tensile strength and tensile fracture energy, at which point the tensile strength reached its minimum. Similarly, Wang et al. examined the flexural properties of carbon fiber/polyimide fiber hybrid composites under different stacking configurations at a constant mixing ratio [23]. Their findings revealed that laminates with the carbon fibers in the outer layers exhibited the highest flexural strength, and that positioning the carbon fibers on the compression side was particularly effective in improving the flexural strength and modulus.

In addition to heat-resistant fibers, high-resistant composites also require a matrix with excellent thermal stability. Thermosetting polyimide resin, owing to its outstanding heat resistance and mechanical performance, serves as a promising candidate for a composite matrix serving in high-temperature environments [24,25]. To the best of our knowledge, only a few reports exist on hybrid fiber composites with a PI matrix, and investigations into their high-temperature (≥300 °C) mechanical behavior remain limited. Such hybrid composites exhibit promising application prospects in high-temperature aerospace fields, such as aero-engine casings, and are therefore worthy of investigation.

In this work, T800-grade carbon fiber and high-modulus, high-strength polyimide fiber were employed as reinforcements, with polyimide resin serving as the matrix. The effects of the hybrid ratio on the tensile, compressive and flexural properties, as well as the failure modes, were systematically investigated. The mechanical properties at room and elevated temperatures were also compared to analyze the hybrid effect. This study aimed to provide new insights into the optimization of the hybrid structure and material selection strategies for the development of heat-resistant and impact-resistant composite structures.

## 2. Materials and Methods

T800-grade carbon fiber (GW800G) was provided by Weihai Guangwei Composites Co., Ltd. (Weihai, China). High-strength and high-modulus PI fiber (S35M, 1700D) was provided by Jiangsu Shino New Materials & Technology Co., Ltd. (Changzhou, China). The mechanical parameters of the fibers were all measured by the authors and are listed in Table 1. A kind of self-developed phenylethynyl-terminated polyimide resin (PI 370) was adopted, whose glass transition temperature is about 450 °C.

**Figure 1:**
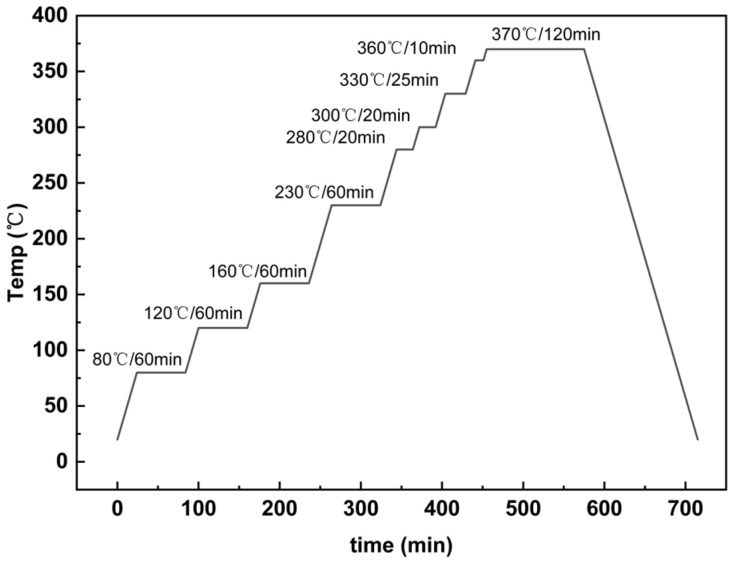
Curing temperature of composite laminate.

Unidirectional PI fiber prepreg (areal weight: 234 g/m^2^; resin content: 50 wt%) and unidirectional carbon fiber prepreg (areal weight: 245 g/m^2^; resin content: 45 wt%) were fabricated using the hot-melt adhesive film method, cut into sheets of 300 × 250 mm, and manually laid up according to the stacking sequences specified in Table 2. The unidirectional laminates were subsequently consolidated and cured using an automatic hot press to produce composite panels with different mixing ratios. The mixing ratio was defined as the volume fraction of carbon fiber relative to the total fiber content, as shown in Equation (1):

(1) $$\mathrm{Mixing}\;\mathrm{ratio}=\frac{V_{C}}{V_{C}\;+{\;V}_{P}}$$

where *V_P_* and *V_C_* are the volume proportion of PI and carbon fiber in the total fiber, respectively, with *V_P_* + *V_C_* = 1. The stacking sequence, given in Table 2, shows a hybrid sandwich structure. The high elastic modulus of the outer carbon fibers facilitates rapid stress wave propagation, while the high toughness of the intermediate PI fibers promotes efficient impact energy absorption. Therefore, in the present study, all hybrid laminates adopt a sandwich structure with carbon fibers as the outer layers and PI fibers as the core. The curing procedure is illustrated in Figure 1. During the 330 °C isothermal stage, cycles of pressurization and depressurization were applied to facilitate the expulsion of small molecules and reduce porosity, after which the pressure was stabilized at 4 MPa until the end of curing. The thickness of all cured laminates was about 2 mm, and there were no obvious defects in these composites, which was confirmed by means of ultrasonic nondestructive testing (Figure S1) and microscopic observation (Figure S2).

The mechanical testing standards for composite laminates in this work are detailed in Table 3. Tensile and compressive properties of the composites were tested on an electronic universal material testing machine (Instron 3382, Instron Co., Ltd, Norwood, MA, USA), while their flexural properties were tested on a universal material testing machine (Instron 8801, Instron Co., Ltd, Norwood, MA, USA). The span-to-depth ratio was 32 for specimens in the three-point bending test. Based on the glass transition temperatures of the PI resin and fibers (approximately 400 °C and 350 °C, respectively) and the criterion that the long-term service temperature is approximately Tg-50~100 °C, flexural tests were conducted at room temperature, 300 °C, and 370 °C. A total of five specimens were tested for each group. The mean values and standard deviations are presented in the corresponding figures. The macroscopic morphology of the specimens was recorded using a digital camera and an ultra-depth optical microscope (VHX-6000, Keyence Co., Ltd, Osaka, Japan). Microscopic morphology after tensile failure was observed on a JSM-IT810 scanning electron microscope (SEM, JEOL Co., Ltd, Tokyo, Japan). Dynamic mechanical analysis (DMA) was conducted using a TA Instruments Q800 (New Castle, DE, USA). The temperature was ramped from 25 °C to 500 °C at a heating rate of 5 °C/min under a nitrogen atmosphere.

## 3. Results and Discussion

### 3.1. Tensile Properties

**Figure 2:**
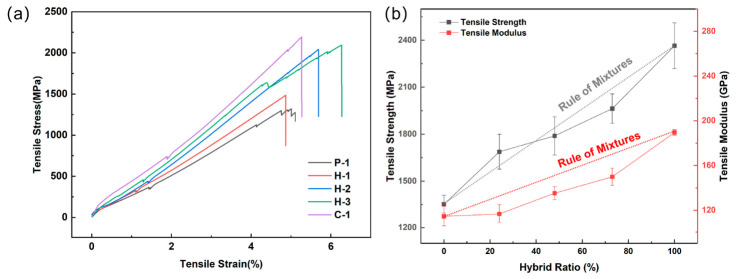
Tensile properties of hybrid composite materials: (**a**) stress–strain curve, (**b**) variation of tensile strength and modulus with hybrid ratio.

Unidirectional composite laminates with different hybrid ratios are subjected to tensile tests along the fiber direction (0°). The resulting tensile stress–strain curves and the tensile performance data are illustrated in Figure 2.

Prior to tensile failure, both the C-1 and hybrid laminates exhibit clear linear characteristics, whereas the P-1 laminate shows multiple stress-drop phenomena near the maximum stress (Figure 2a). The fracture elongations of the carbon fiber and the PI fiber used in this study were 2%; thus, the fracture elongations of the C-1 and P-1 laminates were similar.

Among the hybrid composites, with the exception of H-1 whose fracture elongation is comparable to that of the carbon fiber and PI fiber composites, the other hybrid composites exhibit higher fracture elongation values (H-3 > H-2 > H-1). This can be attributed to the hybrid effect. Although the apparent fracture elongation is similar, carbon fibers are more sensitive to defects and stress concentration. Under tensile loading, some of the weakest carbon fibers break first, whereas the high-toughness PI fibers remain intact, continue to carry the load, and effectively redistribute the stress around the fractured carbon fibers. As the load increases further, the remaining carbon fibers gradually fracture, while the PI fibers, owing to their toughness, continue to elongate until ultimate failure. This sequential fracture process, in which carbon fibers break progressively and the PI fibers ultimately fail last, extends the overall load–displacement curve, resulting in an apparent fracture elongation that is higher than that of the individual materials.

To assess the hybrid effect, the hybrid law was introduced, as shown in Equation (2):

(2) $$P_{H}=P_{P}V_{P}+P_{C}V_{C}$$

In this equation, *P_P_* and *P_C_* represent the corresponding performance parameters (in this paper, strength and modulus) of P-1 and C-1 laminates, while *V_P_* and *V_C_* denote the volume fractions of PI fibers and carbon fibers in the total fiber composition, with *V_P_* + *V_C_* = 1. When the measured value is larger than the calculated value *P_H_*, a positive hybrid effect is considered to occur. Conversely, it is regarded as a negative hybrid effect [20]. All the subsequent mixed laws were calculated using this formula.

As shown in Figure 2b, the tensile strength of the hybrid samples increases with the hybrid ratio, in accordance with the hybrid law. A positive hybrid effect is observed at a hybrid ratio of 24%, whereas negative hybrid effects occur at 48% and 73%. The tensile modulus of the hybrid samples also increases with the hybrid ratio, aligning with the hybrid law and also exhibiting a negative hybrid effect. This behavior can be attributed to the following mechanism. During the initial loading phase, both fibers work synergistically to bear the load. However, due to the mismatch in moduli, the carbon fibers actually experience higher stress than anticipated, which leads to premature fracture of the weakest carbon fibers due to overload. In the later stages of loading, as the carbon fibers fracture progressively and fail prematurely, their full-strength potential is not fully utilized, resulting in a reduction in the overall tensile properties and thus the manifestation of a negative hybrid effect.

**Figure 3:**
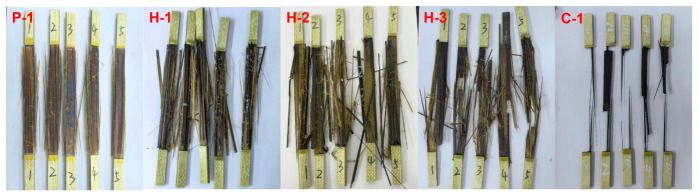
Tensile failure modes of different composite laminates (macroscopic).

Figure 3 shows the macroscopic morphology of the composites after tensile failure. In the hybrid composites, as the hybrid ratio increases, the macroscopic fracture behavior observed after the tensile test gradually transitions from the ductile behavior of P-1 to the brittle behavior of C-1. This change can be attributed to the physical barrier effect of PI fibers and the attenuation of stress wave propagation caused by the modulus mismatch between the carbon fibers and PI fibers. As a result, cracks initiated by the fracture of the carbon fibers do not rapidly propagate throughout the laminate but instead release energy in a more controlled manner. Consequently, most of the carbon fibers remain intact within the hybrid composite after failure, thereby preventing the occurrence of secondary damage.

**Figure 4:**
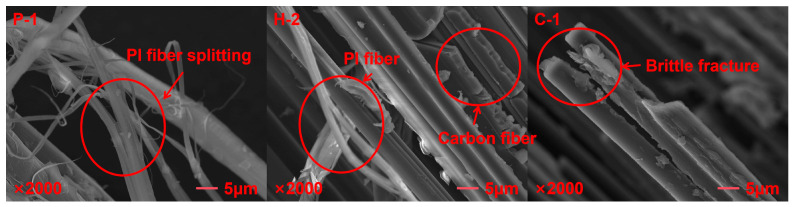
Tensile failure mode of different composite laminates (microscopic).

The post-tensile fracture of different composite laminates was further examined using scanning electron microscopy, as shown in Figure 4. After the fracture of P-1, the PI fibers are relatively dispersed, particularly at the sites of fiber fracture, where the PI fibers exhibit bending, severe deformation, splitting, and fibrillation into microfibers. This behavior results from conformational changes in the molecular chains after yielding, which lead to the formation of a plastic zone and induce chain mobility [20]. In contrast, in the fractured C-1 laminate, the carbon fibers remain straight and tightly bonded with the resin, and their cross-sections clearly display brittle fracture characteristics. The morphology of the hybrid laminate reveals that PI microfibers are attached to the surfaces of the carbon fibers, suggesting the formation of a well-integrated hybrid interface between the two kinds of fibers.

### 3.2. Compressive Properties

**Figure 5:**
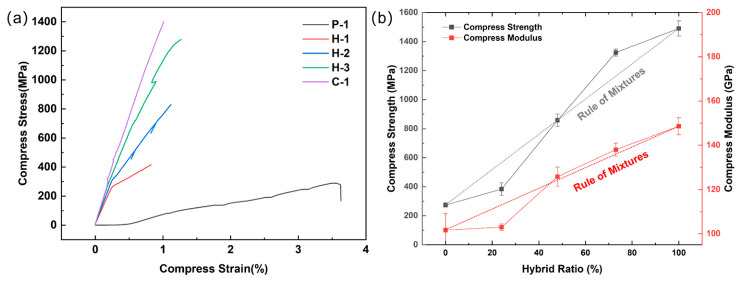
Compression performance of hybrid composites: (**a**) stress–strain curve, (**b**) variation of compressive strength and modulus with hybrid ratio.

Compression tests along the fiber direction (0°) were conducted on P-1, C-1, and hybrid laminates, with their corresponding compressive stress–strain curves shown in Figure 5.

All curves show an immediate decline after reaching the critical compressive stresses, indicating that the material fails instantaneously (Figure 5a). The compressive performance of the P-1 and C-1 laminates differs significantly. As the volume fraction of carbon fibers in the hybrid composite increases, both the strength and modulus of the hybrid compression samples are substantially improved. PI fibers, being inherently tough but having a low modulus, are prone to micro-buckling under compression. Due to the poor lateral support from the low-strength matrix, P-1 undergoes significant post-buckling deformation, thereby exhibiting a larger compressive failure strain. In contrast, the incorporation of high-modulus carbon fibers significantly improves the laminate’s resistance to buckling and restricts deformation, leading to a smaller failure strain for C-1, which instead fails in a more brittle manner. Under compressive loading, the failure of carbon and PI fibers occurs asynchronously; the carbon fibers at weak points initially undergo micro-buckling or shear fracture. However, these micro-cracks are immediately mitigated and passivated by the surrounding tough PI fibers and the resin matrix, a process that consumes a large amount of energy and allows the material to continue bearing load while accumulating further micro-damage. Therefore, the hybrid composite can withstand greater overall deformation before final failure, resulting in an increase in macroscopic compressive strain. As shown in Figure 5b, the compressive strength of the hybrid samples increases with the hybrid ratio, following the hybridization law, and exhibits a positive hybrid effect. The compressive modulus also increases with the hybrid ratio, consistent with the hybridization law, with the exception of the H-1 sample, where the hybrid samples all display positive hybrid effects.

The improvement in compressive strength is primarily attributed to the significant enhancement of the buckling resistance and transverse shear strength due to the carbon fibers. The high rigidity of the carbon fibers makes them more resistant to buckling, while also providing lateral support to the adjacent PI fibers. The incorporation of carbon fibers also increases the overall shear modulus of the laminate, making it less susceptible to failure due to shear deformation. Before reaching the failure load, both fibers effectively share the compressive load, with the carbon fibers bearing the majority of the stress, thereby significantly increasing the maximum load-bearing capacity before macroscopic failure. This results in a positive hybrid effect on the compressive strength. The compressive modulus of the carbon fibers is much higher than that of the PI fibers. During the initial stage of compressive loading, both fibers deform cooperatively within the matrix. According to the hybridization law, the addition of high-modulus carbon fibers effectively enhances the overall compressive modulus of the composite. This is in obvious contrast to the decrease in the modulus observed during the tensile loading due to the interface strain incompatibility of the hybrid composite. For the H-1 sample, which exhibits a negative hybrid effect, the lower proportion of carbon fibers results in the presence of an “ineffective volume” [20], which fails to effectively improve the performance of the hybrid sample.

**Figure 6:**
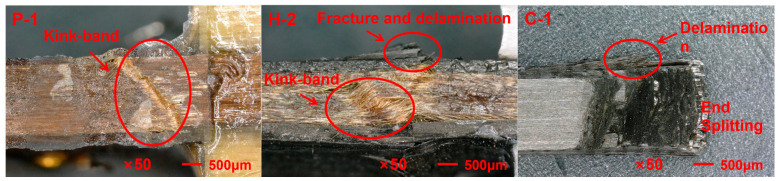
Compression failure modes of different composite laminates.

Figure 6 presents the optical images of these composites after compressive failure. The P-1 compression specimen did not fracture but instead formed a torsion band. The formation of the torsion band may be attributed to defects in the load alignment. As the compressive load increases, the resin matrix undergoes shear yielding, causing debonding at the interface between the matrix and fibers. This results in uneven load transfer, and the matrix no longer provides sufficient lateral stability to the PI fibers, leading to the loss of stability in the PI fibers and the formation of the torsion band. At the ends of the C-1 specimen, failure occurred, including fiber fracture and shear failure. In the hybrid samples, interface failure occurred, with multiple delamination between the PI fiber layer and the carbon fiber layer, as well as within the PI fiber layer itself. Additionally, the carbon fiber layer experienced compressive fracture, and the PI fiber layer exhibited torsion band failure. The unique failure morphology of the hybrid composite arises from the fact that cracks initiated in the carbon fiber layer do not propagate through the PI fiber layer, but instead spread longitudinally at the interface, ultimately leading to delamination. The PI fiber layer in the hybrid composite effectively prevents the onset of brittle failure, thus maintaining the integrity of the specimen.

### 3.3. Flexural Properties

#### 3.3.1. Room Temperature

**Figure 7:**
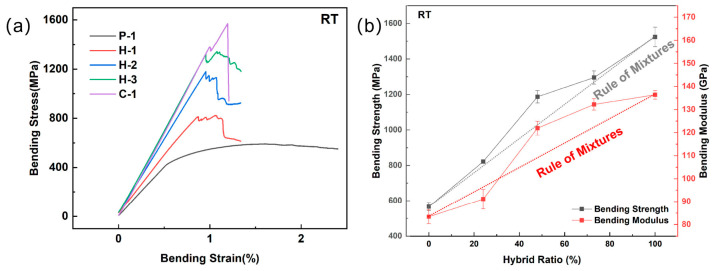
Bending performance of hybrid composites at room temperature: (**a**) stress–strain curve, (**b**) bending strength and modulus variation with hybrid ratio.

Three-point flexural tests were conducted on P-1, C-1, and hybrid laminates at ambient temperature, with the test results shown in Figure 7. Except for P-1, the bending stress–strain curves of the other samples dropped sharply after reaching the maximum point. This behavior mainly results from the inherent high toughness of PI fibers and their distinct failure mechanism compared to carbon fibers. In pure PI fiber composites, significant plastic deformation occurs during the three-point bending test, where the PI fibers do not fracture immediately. Instead, they undergo large-scale, progressive bending in conjunction with the yielding of the resin matrix, creating a load-bearing plateau on the stress–displacement curve. In contrast, in carbon fiber and hybrid composites, brittle carbon fibers, positioned on the top and bottom surfaces of the laminate, bear the majority of the load. When the bending load causes the innermost carbon fibers to reach their compressive strain limit, they buckle instantly, triggering sudden fracture, which results in a sharp stress peak on the curve.

As shown in Figure 7b, the flexural strength of the hybrid samples increases with the hybrid ratio, following the hybridization law and exhibiting a positive hybrid effect. The flexural modulus also increases with the hybrid ratio, consistent with the hybridization law. Additionally, except for the H-1 sample, all other hybrid composites show positive hybrid effects in the flexural modulus.

In the three-point bending test, the stress distribution across the cross-section of the beam is uneven. The upper surface of the sample, near the loading point, experiences the maximum compressive stress, while the lower surface, near the support point, bears the maximum tensile stress. In the hybrid samples, both the top and bottom surfaces are composed of carbon fiber layers, with PI fibers in the center. The carbon fiber layers, which offer the greatest load-bearing capacity, are positioned at the surfaces where the stress is the highest. Therefore, it is reasonable that the bending performance of the hybrid laminates is higher than that predicted by the hybridization law. In contrast, for the H-1 sample, the carbon fiber layers on the top and bottom surfaces are thinner, resulting in minimal enhancement of performance; thus, it does not exhibit a positive hybrid effect.

**Figure 8:**
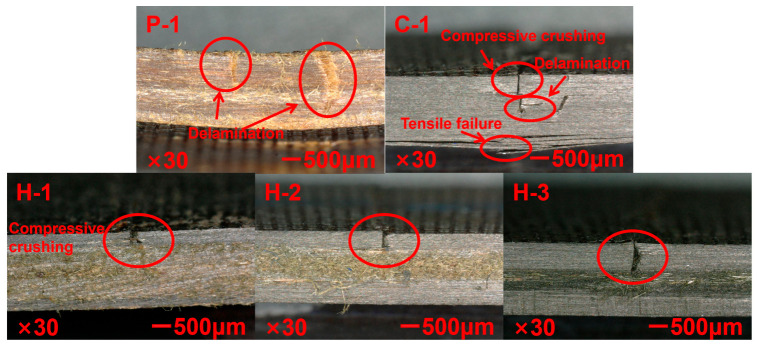
Bending failure modes of different composite laminates at room temperature. The failure modes of composites marked by red circles in Figs. P-1, C-1 and H-1 are described in the accompanying text. Those in Figs. H-2 and H-3 are consistent with Fig. H-1.

**Figure 9:**
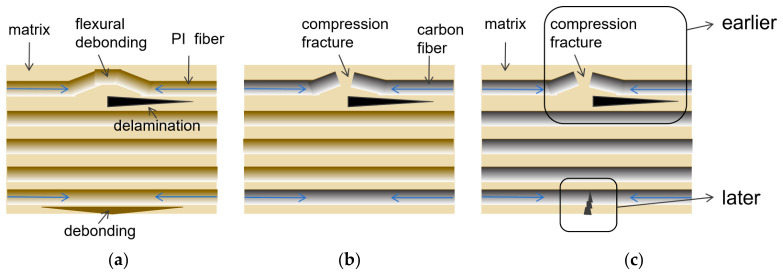
Schematic diagram of the bending failure mode: (**a**) PI fiber composite, (**b**) carbon fiber composite, (**c**) hybrid composite. Blue arrows denote stress directions adjacent to the upper and lower surfaces of the composite in bending tests.

Figure 8 shows the optical images of the composite bending test samples with different hybrid ratios, illustrating the failure modes of the samples. Figure 9 shows the bending failure mode of different composite materials. The bending failure of P-1 primarily manifests as compressive failure of the fibers on the compressed side, as well as tensile failure of the fibers on the tensile side. On the compressed side, the fibers undergo buckling and debonding from the resin. For C-1, the bending failure also includes compressive failure of the fibers on the compressed side and tensile failure of the fibers on the tensile side. However, due to the brittleness of the carbon fibers, the fibers on the compressed side are directly crushed, accompanied by some shear failure. On the tensile side, the fibers break directly under tension, and delamination is observed. The bending samples of the hybrid composites exhibit compressive failure of the inner carbon fibers, along with partial delamination involving the PI fibers. This occurs because the compressive strength of carbon fibers is much lower than their tensile strength. As a result, under bending loads, the hybrid composite first experiences compressive failure of the inner carbon fibers.

#### 3.3.2. High Temperature

**Figure 10:**
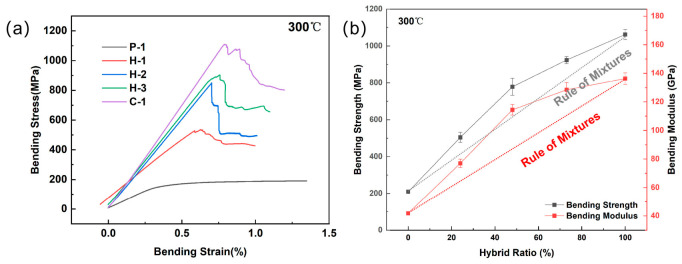
Bending performance of hybrid composites at 300 °C: (**a**) stress–strain curve, (**b**) variation of bending strength and modulus with hybrid ratio.

**Figure 11:**
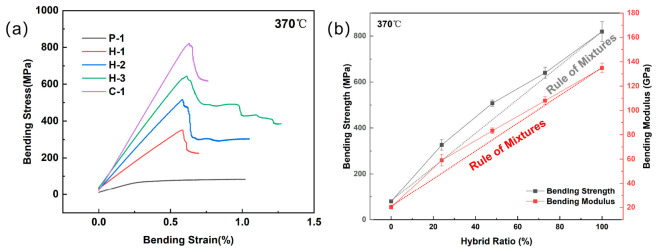
Bending performance of hybrid composites at 370 °C: (**a**) stress–strain curve, (**b**) variation of bending strength and modulus with hybrid ratio.

Based on the operating temperature of aircraft structures and the expected long-term usage temperature of PI resin, high-temperature bending performance tests were conducted at 300 °C and 370 °C, and the test results are presented in Figure 10 and Figure 11.

For P-1, during the initial loading phase, the material undergoes elastic deformation, with the resin and fibers sharing the load. The stress increases with strain, and when the stress reaches a critical value, the organic fibers exhibit some plasticity at high temperatures. After the stress reaches the yield point, the fibers on the compressed side buckle, while the fibers on the tensile side undergo continuous plastic deformation instead of fracture, leading to a plateau on the load curve where the macroscopic load can no longer increase.

In contrast, both the pure carbon fiber composite samples and the hybrid composite samples, where both the compressed and tensile sides consist of carbon fibers, exhibit significant increases in the overall stiffness and strength of the laminate. Their initial modulus and strength are much higher than those of the pure PI fiber laminate.

**Figure 12:**
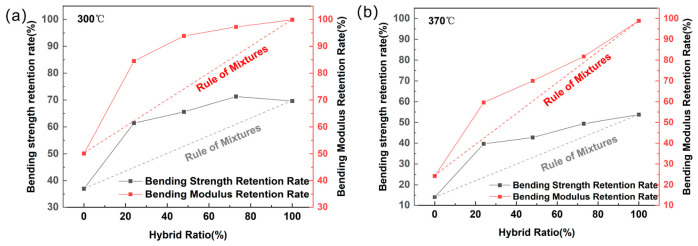
Retention rate of high-temperature bending performance of hybrid composites: (**a**) 300 °C, (**b**) 370 °C.

As shown in Figure 10b and Figure 11b, both the bending strength and modulus of the hybrid laminates increase with the hybrid ratio, in accordance with the hybridization law, and exhibit positive hybrid effects. Furthermore, at 300 °C or 370 °C, the high-temperature performance retention of the hybrid samples also demonstrates obvious positive hybrid effects (Figure 12).

**Figure 13:**
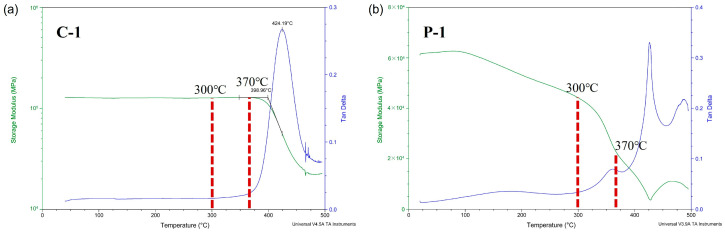
DMA curves of composite: (**a**) C-1, (**b**) P-1.

As shown in Figure 13, the storage modulus of C-1 does not show significant reduction before 370 °C. In contrast, the storage modulus of P-1 decreases significantly with increasing temperature. Due to the higher temperature resistance of carbon fibers, C-1 maintains a relatively high stiffness at elevated temperatures. With carbon fibers on both the upper and lower surfaces of the bending sample, the overall stiffness of the laminate is greatly enhanced, leading to a positive hybrid effect on the bending modulus. Additionally, the presence of carbon fibers on both surfaces significantly improves the compressive strength of the upper surface and the tensile strength of the lower surface, further contributing to a positive hybrid effect on the bending strength. As both surfaces of the bending sample are made of carbon fibers, the regions of maximum load-bearing capacity are entirely occupied by carbon fibers. Therefore, the retention rates of the bending strength and modulus shift toward those of the carbon fiber laminate, resulting in positive hybrid effects in the high-temperature performance retention.

**Figure 14:**
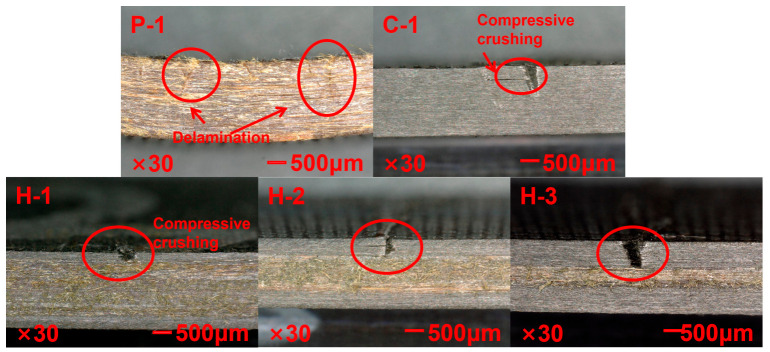
Bending failure modes of different composite laminates at 300 °C. The failure modes of composites marked by red circles in Figs. P-1, C-1 and H-1 are described in the accompanying text. Those in Figs. H-2 and H-3 are consistent with Fig. H-1.

**Figure 15:**
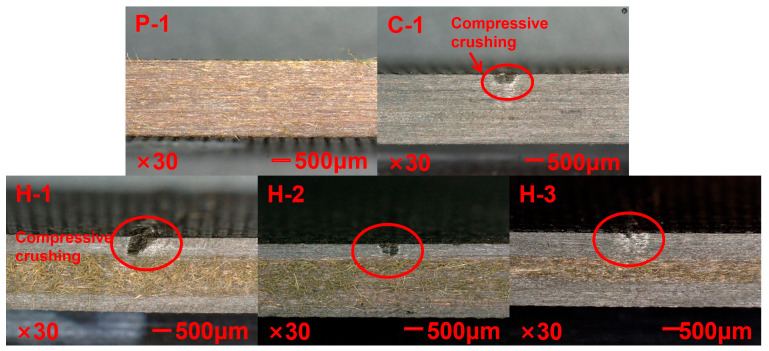
Bending failure modes of different composite laminates at 370 °C. The failure modes of composites marked by red circles in Figs. P-1, C-1 and H-1 are described in the accompanying text. Those in Figs. H-2 and H-3 are consistent with Fig. H-1.

Figure 14 and Figure 15 present the optical images of the composite samples after bending tests at 300 °C and 370 °C. At 300 °C, the bending failure of P-1 primarily manifests as compressive failure of the fibers on the compressed side, along with tensile failure of the fibers on the tensile side. On the compressed side, the fibers undergo buckling and debonding from the resin. At 370 °C, P-1 does not exhibit obvious failure characteristics, which can be attributed to the fact that this temperature exceeds the glass transition temperature of the PI fibers, causing them to transition into a rubbery state. At 300 °C, the bending failure of C-1 also involves compressive failure of the fibers on the compressed side, accompanied by delamination. At 370 °C, the failure is primarily characterized by compressive failure of the carbon fibers. For the hybrid composites, regardless of 300 °C and 370 °C, the bending samples show compressive failure of the inner carbon fibers. This is because the compressive strength of carbon fibers is much lower than their tensile strength. Therefore, under bending loads, the hybrid composites initially experience compressive failure of the inner carbon fibers. Compared with the flexural behavior of the hybrid composite at room temperature, the failure mode under high-temperature conditions shows no significant change, indicating that the addition of carbon fibers enhances the stability of the composite in high-temperature environments.

Based on the experimental results presented above, the carbon fiber/PI fiber hybrid-reinforced polyimide resin composite system not only exhibits excellent mechanical properties but also demonstrates outstanding high-temperature resistance, with superior mechanical property retention under elevated temperature conditions, distinguishing it from other fiber hybrid composites.

## 4. Conclusions

This study focused on the preparation of a series of hybrid composites using high-strength, high-modulus PI fibers and carbon fibers as reinforcements, with high-temperature resistant PI resin as the matrix. The effects of the hybrid ratio on the mechanical properties and failure modes of the composites were discussed. The impacts of fiber hybridization on the tensile, compressive, and bending performances, as well as the failure morphology, were studied. The mechanical properties of the hybrid composites at elevated temperatures were further investigated. The following conclusions were obtained.

The tensile strength and modulus increase with the hybrid ratio. However, a positive hybrid effect is observed only for the tensile strength at the initial loading stage, while negative hybrid effects prevail throughout the remainder of the deformation process. This behavior is attributed to the modulus mismatch between the two fibers, which prevents the carbon fibers from fully realizing their strength potential prior to fracture. The compressive strength and modulus also increase with the hybrid ratio, and the incorporation of carbon fibers significantly enhances the overall strength and stiffness of the composite. The carbon fibers help suppress buckling and provide good support to the PI fibers, ultimately leading to positive hybrid effects.

The bending performances of the hybrid composites, both at room temperature and high temperatures, follow the hybridization law. Regardless of the testing temperatures, the bending performance improves with the increase in the hybrid ratio. Additionally, since the carbon fibers are located on both the upper and lower surfaces of the sample, their compressive and tensile properties are much higher than those of the PI fibers, resulting in positive hybrid effects in the flexural properties. The storage modulus of the carbon fiber composite remains nearly unchanged under high-temperature conditions, thereby greatly enhancing the high-temperature mechanical property retention of the hybrid composite.

In conclusion, rational hybrid structural design can enhance the performance of hybrid fiber reinforced composites by maintaining the stiffness and strength of the carbon fiber composite, while achieving the toughness of the PI fiber composite. This makes them a promising class of high-strength, high-toughness structural composites in high-temperature application fields.

## Figures and Tables

**Table 1:** Properties of the selected fibers.

Fiber Type	Tensile Strength (MPa)	Tensile Modulus (GPa)	Elongation (%)	Density (g/cm^3^)	IFSS (MPa) with PI 370
Carbon fiber (GW800G)	6400	295	2	1.78	72.4
Polyimide fiber (S35M)	2950	178	2	1.46	24.3

**Table 2:** Stacking sequence of hybrid composites.

Laminate Type	Laminate Code	Ply Number Ratio (Carbon Fiber/ PI Fiber)	Stacking Sequence	Mixing Ratio (%)
PI fiber composite	P-1	0/16	○○○○○○○○○○○○○○○○	0
Carbon fiber composite	C-1	16/0	●●●●●●●●●●●●●●●●	100
Hybrid composite	H-1	2/12/2	●●○○○○○○○○○○○○●●	24
	H-2	4/8/4	●●●●○○○○○○○○●●●●	48
	H-3	6/4/6	●●●●●●○○○○●●●●●●	73

Note: ○ represents PI fiber and ● represents carbon fiber; H-1, H-2, and H-3 are the 3 subgroups for HFRP specimens, subgroups for HFRP specimens.

**Table 3:** Testing methods and conditions for mechanical property measurements.

Test Item	Standard	Specimen Dimension (mm)	Loading Rate (mm/min)
Longitudinal tension	GB/T 3354-2014 [26]	250 × 12.5 × 2	2
Longitudinal compression	GB/T 5258-2008 [27]	110 × 10 × 2	1
Three-point bending	GB/T 3356-2014 [28]	80 × 12.5 × 2	2

## Data Availability

The original contributions presented in this study are included in the article/Supplementary Materials. Further inquiries can be directed to the corresponding authors.

## Supplementary Materials

The following supporting information can be downloaded at: https://www.mdpi.com/article/10.3390/polym18111322/s1, Figure S1: Ultrasonic images of five types of composite laminates; Figure S2: Metallographic images, (a) C-1, (b) P-1, (c) H-2.
