# Supplementary material for: Mechanical Properties of Carbon Fiber and Polyimide Fiber Hybrid Reinforced Polyimide Resin Matrix Composites at Room and High Temperatures
Source: Polymers (Basel). 2026 May 27;18(11):1322. doi: 10.3390/polym18111322 (PMC13259207; doi:10.3390/polym18111322)
Supplement: Supplementary file 1 [file polymers-18-01322-s001.zip › polymers-4296776-supplementary.pdf]

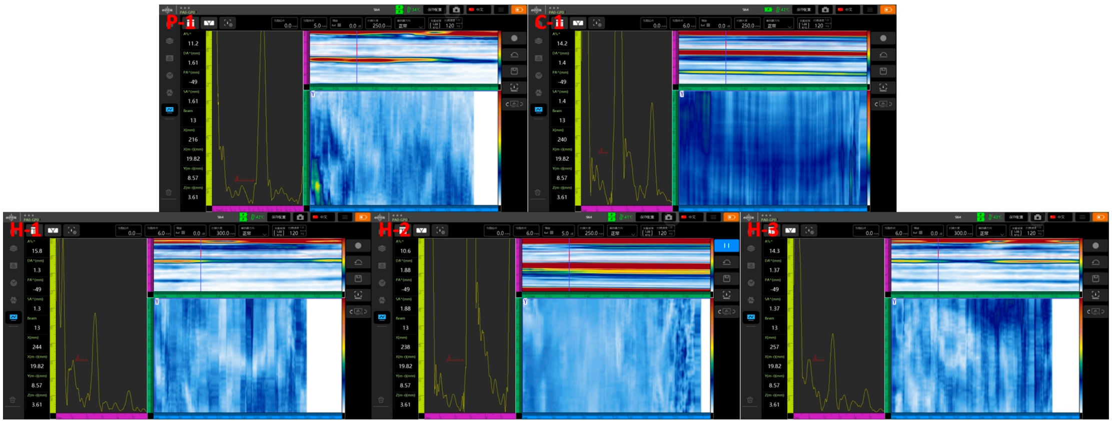

Figure S1. Ultrasonic images of five types of composite laminates.

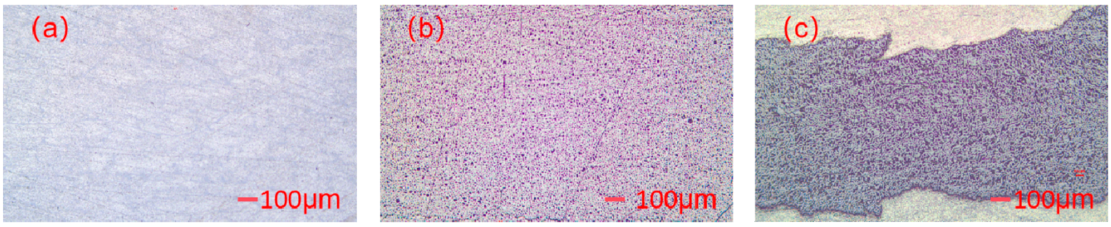

Figure S2. SEM images, (a) C-1, (b) P-1, (c) H-2.

Since the ultrasonic results were satisfactory and the correlation between the ultrasonic results and the metallographic images had been verified, SEM images were not taken for H-1 and H-3.
